# Periodization of Carbohydrate Intake: Short-Term Effect on Performance

**DOI:** 10.3390/nu8120755

**Published:** 2016-11-25

**Authors:** Laurie-Anne Marquet, Christophe Hausswirth, Odeline Molle, John A. Hawley, Louise M. Burke, Eve Tiollier, Jeanick Brisswalter

**Affiliations:** 1 Laboratory of Sport, Expertise and Performance, French National Institute of Sport, Expertise and Performance (INSEP), 75012 Paris, France; hausswirthc@gmail.com (C.H.); odeline.molle@insep.fr (O.M.); eve.tiollier@insep.fr (E.T.); 2 Université Côte d’Azur, LAMHESS, 06205 Nice, France; brisswalter@unice.fr; 3 Mary MacKillop Institute for Health Research, Centre for Exercise and Nutrition, Australian Catholic University, Melbourne, VIC 3065, Australia; John.Hawley@acu.edu.au (J.A.H.); louise.burke@ausport.gov.au (L.M.B.); 4 Research Institute for Sport and Exercise Sciences, Liverpool John Moores University, Liverpool L3 5UA, UK; 5 Sports Nutrition, Australian Institute of Sport (AIS), Belconnen, ACT 2617, Australia

**Keywords:** carbohydrate, performance, training, cycling time trial, trained athletes, lipid oxidation, perception of effort

## Abstract

Background: “Sleep-low” consists of a sequential periodization of carbohydrate (CHO) availability—low glycogen recovery after “train high” glycogen-depleting interval training, followed by an overnight-fast and light intensity training (“train low”) the following day. This strategy leads to an upregulation of several exercise-responsive signaling proteins, but the chronic effect on performance has received less attention. We investigated the effects of short-term exposure to this strategy on endurance performance. Methods: Following training familiarization, 11 trained cyclists were divided into two groups for a one-week intervention—one group implemented three cycles of periodized CHO intake to achieve the sleep-low strategy over six training sessions (SL, CHO intake: 6 g·kg^−1^·day^−1^), whereas the control group consumed an even distribution of CHO over the day (CON). Tests were a 2 h submaximal ride and a 20 km time trial. Results: SL improved their performance (mean: +3.2%; *p* < 0.05) compared to CON. The improvement was associated with a change in pacing strategy with higher power output during the second part of the test. No change in substrate utilization was observed after the training period for either group. Conclusion: Implementing the “sleep-low” strategy for one week improved performance by the same magnitude previously seen in a three-week intervention, without any significant changes in selected markers of metabolism.

## 1. Introduction

Carbohydrate-based fuels (CHO) are the main substrates used by the brain and skeletal muscle during exercise. Thus, nutritional recommendations for competition performance promote strategies to achieve “high CHO availability”, in the form of adequate pre-exercise glycogen concentrations and additional CHO intake during competition to meet the specific fuel needs of the event [1,2]. However, recent research has provided new insight into the interactions of exercise with “low CHO availability”, whereby the adaptive responses to training or recovery are enhanced in an environment of low exogenous and endogenous CHO stores [3]. Within this framework, glycogen is not only considered as an energetic substrate, but more as a regulator of metabolic signaling responses [4]. 

The aim of training is to act as a chronic stimulus leading to physiological adaptations and an improvement in performance. The acute and chronic effect of endurance exercise on metabolic responses have already been widely described and include mitochondrial biogenesis, shifts in fiber composition toward type I fibers, and enhanced oxidative metabolism [5,6]. Substrate availability interacts with the contractile stimulus to modulate these physiological responses to training [7] Specifically, muscle glycogen content can modulate physiological adaptations induced by endurance training by upregulating transcription factors and regulators of gene expression such as *PGC-1α* [8] and *p53* [9]. Based on these observations, a growing interest in training under conditions of low glycogen availability and/or low exogenous glucose availability has developed [3].

Several studies have reported that commencing a training session with low glycogen availability enhances expression of genes involved in mitochondrial biogenesis and substrate metabolism [10,11,12,13]. However, these studies have typically failed to show improvements in performance, likely because the beneficial “molecular” effects are negated by a decreased ability to sustain high intensity exercise under the conditions of low CHO availability [12,13]. This has led to interest in a “periodized” approach to CHO availability in the training program, where sessions undertaken to promote adaptation are carefully integrated with others focused on high quality performance outcomes. The “sleep-low” (SL) strategy represents one such sequence of periodized CHO availability, which allows athletes to perform high intensity training sessions supported by high CHO availability while enhancing metabolic adaptation associated with low glycogen availability [14,15,16,17].

Specifically, this strategy consists of a cycling of (1) late afternoon scheduling of a high intensity training (HIT) session undertaken with high glycogen stores; (2) withholding of the ingestion of CHO after the session to maintain glycogen depletion during the overnight recovery period; and (3) a low–moderate intensity steady-state exercise session (LIT) in the following morning completed after an overnight fast. Previous studies have reported that this strategy leads to increased activity of several proteins with putative roles in training adaptation (AMPK, p38 MAPK, p53) [9,14] and higher rates of fat oxidation during submaximal exercise [14]. However, the effects on endurance performance are equivocal. Recently, we [15] reported that integrating SL strategy, three times a week, during a three-week training intervention (i.e., nine occurrences of the sequence) was associated with an improved endurance performance in well-trained subjects (+3% during a 10 km running trial), coupled with an increase in submaximal cycling efficiency. A control group, who undertook the same training program with a similar total intake of energy and CHO, but normally distributed over the day, failed to improve performance. Furthermore, the performance improvements achieved by the SL program were associated with a decrease in body fat (−1.05%) [15] without any negative impact on immune function or sleep quality [16]. The original concept underlying this strategy is the periodization of the CHO intake: instead of a chronically low CHO intake, which has been shown to alter glycogen metabolism [18], high-intensity training sessions are performed under conditions of high glycogen availability. The recovery period, which plays a central role in the development of training adaptation [19], is non optimal for prolonging the period of optimized response to the training stimulus [20]. Lower-intensity training (LIT) is performed while fasted to maximize cellular adaptations and enhance rates of lipid oxidation.

Although the intervention in our three-week study was successful in improving performance and body composition [15], we note challenges to the feasibility of free-living athletes achieving the required dietary manipulations and/or having the commitment to undertake the low CHO recovery and subsequent training [21]. It is therefore of interest to see if a shorter exposure to this CHO periodization strategy would be successful in inducing metabolic adaptations and performance improvement. Accordingly, the aim of the current study was to investigate the effect of an abbreviated program of the “sleep-low” strategy on endurance performance in well-trained athletes. We also examined whether any observed effects on performance are related to an enhancement of metabolic adaptations to training as previously suggested [3].

## 2. Materials and Methods 

### 2.1. Study Population

Eleven endurance-trained male cyclists volunteered to participate in the study. They were healthy, aged between 18 and 40 years, and training at least 12 h/week, having at least 3 years of prior training. Their mean (±SD) age was 31.2 ± 7.1 years, their mean body mass was 71.1 ± 5.6 kg, their mean maximal oxygen consumption ($\dot{V}$O_2max_) was 64.2 ± 6.0 mL·min^−1^·kg^−1^, and their mean maximal aerobic power (MAP, W) was 342 ± 38.3 W. Before entering the study, all participants were examined by a cardiologist to ensure they did not present with abnormal electrocardiograph pattern or contraindications to physical activity. The study’s protocol was approved by local Ethic Committee 2015-AO1136-43 (Paris IDF X, France). After written and verbal explanation, all participants provided their written informed consent to participate.

### 2.2. Study Design

**Figure 1:**
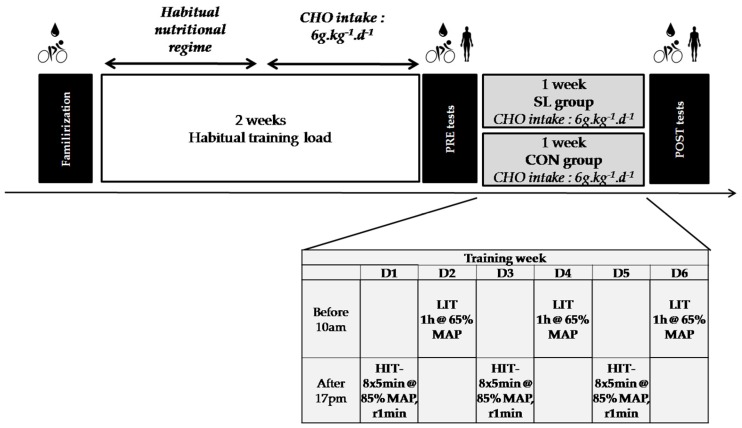
Overview of the experimental protocol; CHO: carbohydrates; HIT: high-intensity training session; LIT: light intensity training session; SL: Sleep-Low; CON: Control; MAP: Maximal aerobic power.

An overview of the study design is depicted in the Figure 1. Subjects were first assigned to a familiarization session to the testing protocol. Then, during the following two weeks, they trained according to their habitual training program. During the first week, they ate according to their usual dietary habits, documenting their food intake via a daily food diary. In the second week, they followed specific nutritional guidelines, which set their CHO intake at 6 g·kg^−1^·day^−1^, while continuing to keep their daily food diary. After the two weeks of habitual training load, subjects were assigned to the PRE test session. Then, they were randomly assigned to two different groups undertaking the same one-week training program but following different nutritional guidelines, according to the “sleep-low” strategy, previously described [14,15]. CHO intake was similar between groups (6 g·kg^−1^·day^−1^) but periodized differently over the day, according to the demands of the training sessions. Specifically, one group trained with a high CHO availability (control group, CON group, *n* = 9) with an even spread of CHO intake over the day and between training sessions. Meanwhile, the intervention group trained with a CHO intake that was periodized within the various days (“sleep-low” group, SL group, *n* = 12) such that no CHO was consumed between the high intensity interval training sessions (HIT) held late in the day and the end of the following morning’s low–moderate intensity (LIT) training session. The protocol ended with a POST test session.

Since it was not possible to disguise the differences in dietary intake between the groups, this study could not be performed as a blinded intervention. In order to limit this bias, participants were not informed of the aim of the study (periodization of the CHO intake). They were neither aware of the number of groups in the study, the group to which they had been assigned, nor the program of the other group.

### 2.3. Preliminary Measurement of Maximal Oxygen Consumption

Before entering the study, all participants had to perform a $\dot{V}$O_2max_ test, which was determined by an incremental test until exhaustion, on an electrically braked cycle ergometer (Excalibur Sport, Lode, Groningen, The Netherlands). Saddle and handlebar heights were set to match the usual positions used by participants, and these were standardized between sessions. The cycle ergometer was equipped with individual racing pedals, allowing participants to wear their own shoes. Subjects warmed up for 6 min at 100 W, then power output was increased by 25 W each successive 2 min until volitional exhaustion. Participants wore a face mask covering their mouth and nose to collect breath (Hans Rudolph, Kansas City, MO, USA). During the test, oxygen uptake ($\dot{V}$O_2_), carbon dioxide uptake ($\dot{V}$CO_2_), minute ventilation ($\dot{V}E$) and the respiratory exchange ratio (RER) were continuously recorded and monitored as breath-by-breath values (Quark, Cosmed, Rome, Italy). The gas and flow analyzers were calibrated prior to each test using ambient air, known-concentration gas (O_2_ = 16%, CO_2_ = 5%), and a 3 L syringe. The $\dot{V}$O_2max_ was determined based on the highest 30 s average value. The MAP (W) was calculated as MAP = W completed + 25 × (t/120), where W is the last completed workload and t is the number of seconds in the last workload [22]. The MAP was used to adjust the workload in the testing session and the training program.

### 2.4. Training Protocol

The training program was divided in two phases. The first phase, lasting two weeks, was composed of the participants’ habitual training programs. The second phase lasted one week and was similar for all participants, regardless of the nutritional group to which they were assigned. The training program (Figure 1) was based on our previous studies [15,16] and consisted of six training sessions over six consecutive days, including a HIT session in the afternoon (after 1700 h) and low–moderate intensity training session in the following morning (before 1000 h). The HIT session comprised a 10 min warm-up followed by eight repetitions lasting 5 min at 85% of MAP interspersed with 1 min of recovery (100 W). The cycling LIT sessions consisted of a steady-state 1 h session at 65% of MAP.

### 2.5. Nutritional Protocol

During the first week of the protocol of the habitual training load, participants were not assigned to specific nutritional guidelines. They were asked to complete a food diary in order to record their nutritional habits and examine how they differed from the nutritional interventions applied in the study. The second week of this first phase of the protocol, all participants were given dietary prescriptions, setting CHO intake at 6 g·kg^−1^·day^−1^ in anticipation of the nutritional strategy of the second phase. Participants were given precise instructions for the weighed food allowances for each meal (breakfast, lunch, dinner, and during training) according to their body mass. During the week of modified training program, participants were separated into two groups: the CON group (*n* = 9) and the SL group (*n* = 12). They were instructed to ingest the same amount of CHO during the day (6 g·kg^−1^) but spread differently over the day (Table 1). A full description of the dietary program can be found elsewhere [16]. Briefly, for the SL group, no CHO was consumed from the commencement of the HIT session on the evening of one day until after the completion of the LIT session on the following morning. Thereafter, CHO intake was resumed to meet daily targets. In this way, the HIT session was undertaken with high muscle glycogen concentrations (“train-high”), while recovery from this session and the completion of the LIT session was undertaken with low CHO availability due to depleted glycogen concentrations and an overnight fast (“sleep-low” and “train-low”, respectively). Meanwhile, high glycogen availability was maintained in the CON group with regular intake of CHO at all meals throughout the day, and the intake of a sports drink (6% CHO, Gatorade, PepsiCo, Purchase, NY, USA) during training sessions. CHO was ingested at every meal. Each participant received written nutritional recommendations for each meal with quantities according to their group and weight. To prevent an unwanted loss of fat-free mass, a high-protein sugar-free drink (High Protein 15 g, UHS, Bruno, France) was prescribed before going to bed. To check compliance to the dietary protocols, participants were required to complete a daily food diary. They were instructed to give as many details as possible (food weights, pictures of dishes, descriptions of fat used to cook or flavor dishes, and the brand names of commercial food products). The diaries were inspected by the same researcher and analyzed using a self-made database of food composition.

Meals during the 24 h prior to the testing sessions (lunch, dinner, and breakfast) were identically prescribed for both groups to ensure that the same amount of CHO (total intake of 6 g·kg^−1^·day^−1^) was consumed.

### 2.6. Testing Protocol

Three sessions of testing were planned: familiarization, PRE, and POST tests. They were composed of two exercise sessions on the same day. The day after the last training session of the week, subjects reported to the laboratory at a standardized time. The first test was a 2 h submaximal cycling test at 60% of MAP at a self-selected cadence. The test started with 10 min at 100 W followed by 110 min at 60% of MAP. Participants wore a cardio belt to monitor heart rate (HR) constantly throughout the test, as well as a face mask to measure gas exchange. They wore the mask for the first 20 min and then the mask was removed for 10 min every 10 min, allowing the subjects to drink only water. Respiratory gases were collected and analyzed to assess cycling efficiency, substrate oxidation, and respiratory quotient. Specifically, whole body rates of CHO and fat oxidation (in g·min^−1^) were calculated from $\dot{V}$O_2_ and $\dot{V}$CO_2_ values measured during the submaximal cycling test; calculations were made from gases collected during the last 60 s of each work interval of interest with nonprotein respiratory exchange ratio (RER) values being assessed according to standard equations [23]:

(1) $$\mathrm{CHO}\;\mathrm{oxidation}=4.210\dot{V}CO_{2}-2.962\dot{V}O_{2}$$

(2) $$\mathrm{Fat}\;\mathrm{oxidation}=1.695\dot{V}O_{2}-1.701\dot{V}CO_{2}$$

Three blood samples were collected during the submaximal test—immediately before, at 1 h, and at 2 h of the test—from a superficial forearm using venipuncture techniques. Four 33 mL samples of blood were collected into EDTA and Z Serum Clot Activator tubes (Greiner Bio-One, Frickenhausen, Germany).

The submaximal test was immediately followed by a 20 km time-trial (TT) performed on the participants’ own bike mounted on a braked Cyclus2 ergometer (RBM GmbH, Leipzig, Germany). We tried to reproduce realistic conditions of a cycling race, within a laboratory environment. Ingestion of sports drink (6% CHO, Gatorade, PepsiCo, Purchase, NY, USA) was allowed during the time-trial, with the volume ingested during the familiarization being recorded and replicated during the ensuing testing sessions. No feedback was provided to the subjects during TT except for their gear ratio and the distance remaining. Rating perception of effort (RPE) was assessed verbally using the Borg 6–20 scale [24] every 5 km. Heart rate (HR) was continuously sampled every 5 s (Polar, Kempele, Finland) during the TT. The time, the mean power, and the mean speed were collected at the end of the TT. Pacing strategy was reported per kilometer during the TT. Participants were not informed of their results until the end of the study.

### 2.7. Blood Analysis

To avoid interassay variation, all blood samples were analyzed in a single batch at the end of the study. Blood samples were collected to measure plasma concentrations of markers of lipid metabolism (glycerol and free fatty acid) and markers of metabolic stress (adrenaline and noradrenaline). After collection, blood samples were immediately centrifuged at 4000 rev·min^−1^ for 10 min at 4 °C to separate plasma from red blood cells. Plasma was then stored in multiple aliquots (Eppendorf type, 1500 µL) at −80 °C until analysis. Catecholamine concentrations were determined with commercially available ELISA kits (Demeditec Diagnostics GmbH, Kiel, Germany). The assay for (adrenaline) had an intra-assay coefficient of variation (CV) of 24.7%–11.0% over a concentration range of 64.7–948 pg·mL^−1^ and an interassay CV of 14.5%–13.1% over a concentration range of 76.4–771 pg·mL^−1^. The assay for noradrenaline had intra-assay CV of 12.8%–11.1% over a concentration range of 510–3363 pg·mL^−1^ and an interassay CV of 9.2%–9.2% over a concentration range of 445–3283 pg·mL^−1^. All blood samples were analyzed in duplicate in respective wavelengths on a spectrophotometer Dynex MRXe (Legalla Biosciences, Chelmsford, MA, USA).

Plasma non-esterified fatty acids (NEFA) were determined with an enzymatic method (Wako Chemical, Neuss, Germany) and glycerol concentrations were measured with enzymatic colorimetric method Randox (Crumnil, Antrin, UK) on PENTRA 400 Horiba (ABX, Montpellier, France).

### 2.8. Body Composition

Measurement of whole body composition was undertaken on all subjects using dual-energy X-ray absorptiometry (Lunar IDXA, General Electric, Madison, WI, USA) at PRE and POST test sessions, the day after the performance tests. All measurements were taken early in the morning and in a fasted state [25].

### 2.9. Statistical Analysis

All statistical analyses were conducted using Statistica 7.1 software (StatSoft). All data are expressed as mean ± SD. Normality of data was tested using a Shapiro–Wilk normality test. Data which were not normally distributed were log-transformed. A repeated-measures analysis of variance (ANOVA) was used to calculate the effect of the dietary strategy (SL vs. CON) and the period (PRE and POST) on performance, blood parameters, and body composition. When a significant effect was found, post hoc tests were performed using Newman–Keuls procedures. Effect sizes for comparison were then calculated Cohen’s d values. Values of 0.1, 0.3, and over 0.5 were respectively considered as small, medium, and large effect [26]. For all tests, the significance level was set at *p* < 0.05.

## 3. Results

### 3.1. Dietary Intervention

Analyses of food diaries revealed that participants complied with the nutritional guidelines of their prescribed diet (Table 1). There was no significant difference in the CHO intake between both groups before and after the training/diet intervention week, despite a slightly difference in the effective CHO intake. Total protein intake increased between the baseline training period and the training diet week (+36.3% and +20.4%, *p* < 0.05, d = 3.48 and d = 1.07, for SL and CON groups, respectively) but without any difference between groups. In both groups, there was also a reduction in reported intake of fat during the training/diet intervention period compared with baseline (−17.8% and −20.9%, *p* < 0.01, d = 2.13 and d = 2.81 for SL and CON groups, respectively).

### 3.2. Performance Tests

#### 3.2.1. Twenty Kilometer Time-Trial Cycling Test Performance

**Figure 2:**
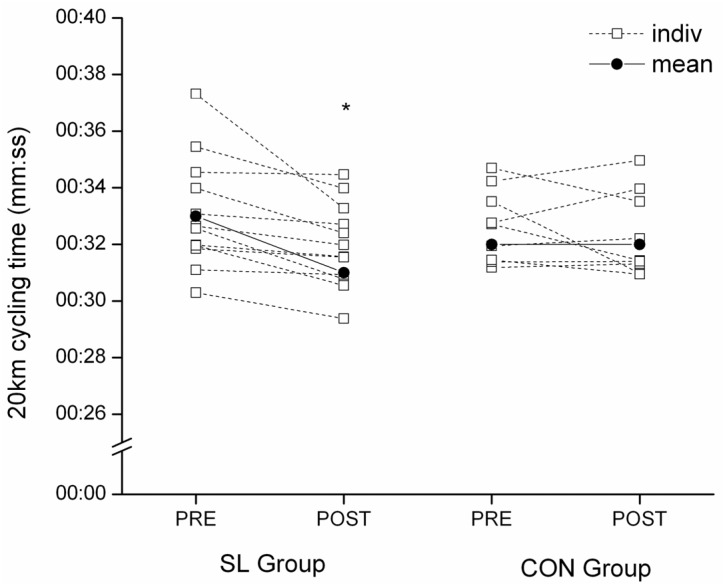
Individual 20 km cycling time-trial performance for SL and CON groups in PRE and POST tests. * Significantly different from PRE values, *p* < 0.05.

Time to complete the 20 km cycling time-trial was reduced after the training period for all the subjects in SL (−3.23% ± 2.99%, *p* < 0.05, d = 1.58), whereas no change was recorded for CON (−1.04% ± 3.46%) (Figure 2). This improvement was due to a significantly higher mean power output (from 229 ± 36 to 250 ± 32 W, *p* < 0.05, d = 1.48) in SL. 

##### • Pacing strategy

**Figure 3:**
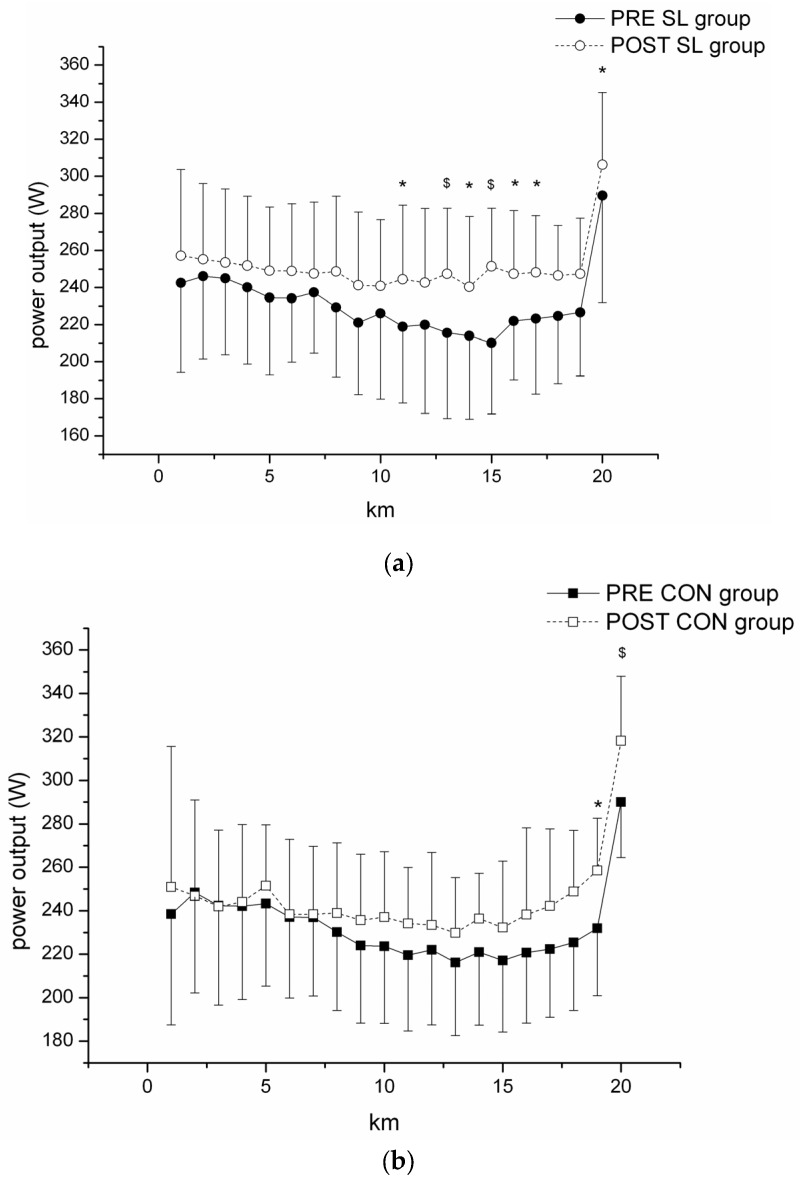
Pacing strategy (absolute change in power output per kilometer) during the 20 km cycling time-trial in PRE and POST tests for (**a**) SL group; and (**b**) CON group. * Significantly different from PRE values, *p* < 0.05. ^$^ Significantly different from PRE values, *p* < 0.01.

The change in mean power over the duration of the time-trial is depicted in Figure 3. The SL strategy induced a significantly higher mean power at the 11th (+13.2% ± 15%, *p* < 0.05, d = 1.58), 13th (+18.1% ± 23.4%, *p* < 0.01, d = 1.95), 14th (+14.3% ± 14.6%, *p* < 0.05, d = 1.58), 15th (21.2% ± 12.8%, *p* < 0.01, d = 2.95), 16th (+11.8% ± 8.4%, *p* < 0.05, d = 1.92), and 17th kilometers (+12.4% ± 9.4%, *p* < 0.05, d = 1.74) (Figure 3a), whereas no change was observed after the training week for the CON group (Figure 3b). Both groups developed higher mean power at the 20th kilometer after the training week (+7.7% ± 14%, *p* < 0.05, d = 0.85 for SL group; +11.2% ± 20%, *p* < 0.01, d = 2.31 for CON group).

##### • RPE

No difference in RPE values during the time trial was observed between PRE and POST tests for both groups (Table 2), despite the higher outputs of the SL group in the POST test trial. 

#### 3.2.2. Submaximal Cycling Test

##### • Substrate oxidation

No significant differences between group and pre and post tests was observed for rates of CHO oxidation (mean values during the whole test: respectively for pre and post test for the SL group 2.0 ± 0.2 g·min^−1^ vs. 2.1 ± 0.2 g·min^−1^; and for the CON group: 1.9 ±0.5 g·min^−1^ vs. 2.1 ± 0.5 g·min^−1^) or fat oxidation (respectively for pre and post test for the SL group 0.6 ± 0.3 g·min^−1^ vs. 0.9 ± 0.2 g·min^−1^; and for the CON group: 0.7 ± 0.2 g·min^−1^ vs. 0.6 ± 0.2 g·min^−1^).

##### • Blood analysis

**Markers of lipid metabolism.** Plasma concentrations of glycerol increased during the submaximal cycling test (*p* < 0.001) but differences between groups or between PRE and POST tests were not significant (Table 3). Similarly, there was an increase in plasma concentrations of free fatty acids during the test (*p* < 0.001) but without any difference between groups or between PRE and POST tests.

**Markers of stress**. Plasma catecholamine concentrations increased during the submaximal cycling test: the concentrations at 1 h and at 2 h were higher than resting concentrations for both groups (*p* < 0.01 for both markers). No significant difference in plasma catecholamine concentrations were observed before and after the training/diet intervention or between groups (Table 3).

### 3.3. Training Period

The perception of effort for the LIT training session during the intervention was significantly different between groups. Subjects who trained in a fasted state (SL group) perceived the LIT training sessions as harder (15.2 ± 1.9) than the subjects of the CON group (13.5 ± 2) (*p* < 0.05, d = 0.87).

### 3.4. Body Composition

There were no differences in body mass and fat-free mass for either group after the intervention week. However, there was a significant reduction in fat mass in the SL group only (−395 ± 491 g, *p* < 0.05, d = 0.34), whereas the change observed in the CON group was not significant (−151 ± 363 g).

## 4. Discussion

This study investigated the effect of a short-term exposure to a periodized “sleep-low” training/diet strategy on metabolism and performance of well-trained cyclists. The program involved exposure to three cycles of a sequence involving “train high, sleep low, and train low” based on periodizing CHO intake to achieve different levels of CHO availability for specific training sessions within a week of training. The main finding was a significant improvement in performance during a cycling time-trial after only one week of training under the “sleep-low” strategy (+3.2% ± 2.99%). This improvement is similar in magnitude to that observed previously after three weeks of SL training [15]. No significant effect was observed for any other physiological parameter. This enhanced performance was related to differences in pacing strategy, and higher levels of self-chosen power outputs in the athletes who undertook the periodized CHO intake protocol. These findings show the importance of pacing in the determination of performance, and suggest factors other than physiological or metabolic characteristics that have been previously reported in studies focusing on the effect of low glycogen availability during training [7].

Strategies that promote training adaptation with low CHO availability (overnight-fasted training, low-glycogen training, low glycogen recovery periods) are commonly observed among athletes, but are often implemented unintentionally or without strategy. The lack of efficacy of these protocols in some studies [12,13] suggests that unless they are implemented in a strategic way, the outcomes may not integrate with other aspects of the training program towards a clear performance improvement. A case study describing the real-life training program of three elite marathoners during a 16-week training program [21] illustrated a sophisticated approach to mixing and matching specific training sessions with varying CHO availability, with the frequency of low CHO training varying from 1.3 to 2.6 sessions/week of training at different times of the season. Our protocol involves a specific sequence of three different training/nutrient stimuli, and this study brings new information regarding how they might achieve benefits in a shorter period or be scheduled at a strategic time before competition [27], at least in athletes of this well-trained but sub-elite caliber. 

The improvement in performance in the current study was associated with change in the pacing strategy. Among the participants in our group who undertook the “sleep-low” exposure, self-chosen power outputs in the second half of the time-trial (11th–17th kilometer) were higher despite the same perceived exertion. Factors affecting pacing strategies have been widely investigated during the last decade and several models have been proposed [28,29,30,31].

It has been suggested that endurance performance is centrally regulated by both intrinsic (cognitive, mental fatigue, physiological) and extrinsic (environmental) signals to preserve physiological limits [32]. In the psychobiological model of Marcora [31], pacing regulation could be explained using an effort-based decision-making model based on motivational intensity theory. This model states that the conscious regulation of pace is determined by five cognitive factors: (1) perception of effort; (2) potential motivation; (3) knowledge of the distance/time to cover; (4) knowledge of the distance/time remaining; and (5) previous experience of perception of effort during exercise of varying intensity and duration. In most of the cases, perception of effort is the key determinant of these models. In any event, the pacing strategy is adopted very rapidly, meaning that it is not only a function of metabolic changes [30]. 

One hypothesis to explain the impact of the periodization of CHO intake on the improvement of performance could reside in changes in resting muscle glycogen concentration. In a twice-daily training model in which the second session was undertaken with low glycogen availability, Hansen et al. and Yeo et al. [11,12] found a higher resting glycogen content in muscle that had received this exposure. It is possible that the participants in the SL group achieved an enhancement of glycogen storage leading to higher muscle glycogen concentration at the start of the 20 km time-trial. Muscle glycogen depletion, when the athlete is fed, is correlated to the development of fatigue [33]. The lower values of RPE after the training period can also be explained by higher muscle glycogen concentration. Rauch et al. [34] proposed that the power output developed is dependent on the brain, which anticipates the rate of muscle glycogen utilization leading to individual “critical” levels of endpoint muscle glycogen. In their study, eight subjects followed three days of carbohydrate loading or a normal diet with an exercise protocol in which they completed 2 h cycling at 65% of MAP interspersed with five 60 s sprints after 20, 40, 60, 80, and 100 min. This bout was followed immediately by a time-trial of 1 h. Although the power outputs developed in the trial following the normal diet were lower than those in the carbohydrate loading trial, endpoint muscle glycogen concentrations were similar in both conditions, despite different starting concentrations. Although we were unable to measure muscle glycogen in our study, it is possible that higher pre-exercise muscle glycogen concentrations in the SL group may “signal” to the brain to allow higher power output. Future studies should investigate this hypothesis.

One limitation of our study which could also explain the possibly higher muscular glycogen content is the trend for an increase in energy and CHO intake for the SL group between PRE and POST testing sessions, while it was slightly reduced for the CON group. We note that although we provided precise nutritional guidelines to participants, they were free-living and prepared their own meals. Therefore, slight deviations from the desired dietary control could have possibly induced a bias in the outcomes. It should be noted, however, that despite these trends in reported energy intake, the SL group reported a small decrease in fat mass over the intervention period. 

Another interesting finding of our study is that the performance improvement seen in the SL group was not associated with the metabolic changes classically reported after training with low CHO availability [14]. No changes in fat oxidation were observed during the submaximal cycling bout in the SL participants, while blood analyses also failed to record any change in metabolites or catecholamine levels after one week of “sleep-low” training strategy. The lack of any effect of the SL strategy on substrate oxidation is similar to the findings of our first study using a three-week SL strategy [15], but contrasts with the observations from previous studies on training with low glycogen availability. Typically, these studies report higher activity of enzymes involved in fat metabolism [12,13], and changes in transcription for adaptive genes [14] or factors involved in mitochondrial biogenesis [17]. However, a difference between our study and others is that our performance tests were undertaken pre- and post- intervention with subjects following strategies of high CHO availability (i.e., high CHO diet in the preceding day, pre-exercise CHO intake, CHO intake during the exercise). Thus, previous studies reported the effect of exercise in fasted conditions [10,35] as well as the effect of training with low CHO availability. In terms of effects on catecholamine concentrations, the lack of changes in the current study are consistent with the findings of our longer study, in which an increase in resting catecholamine concentrations was observed in the second and the third week of the training/diet intervention. This indicates that a longer period of exposure is needed to achieve measurable modifications in plasma catecholamine concentration.

## 5. Conclusions 

One week of training with sequential periodization of CHO availability for selected periods of training (recovery, light intensity training session) seems sufficient to improve performance in trained endurance athletes. This strategy could be implemented during the weeks preceding a competition before the taper period. 

## Figures and Tables

**Table 1:** Total energy and macronutrient intake for sleep-low (SL) and control (CON) groups before starting the training program (BASELINE) and during the training/diet intervention (TRAINING) (mean ± SD).

		Total Energy Intake	Carbohydrate Intake	Lipid Intake	Protein Intake
		(kcal·Day^−1^)	(g·kg^−1^·Day^−1^)	(g·kg^−1^·Day^−1^)	(g·kg^−1^·Day^−1^)
**SL group**	BASELINE	2658 ± 726	4.9 ± 1.3	1.2 ± 0.4	1.4 ± 0.4
*n* = 12	TRAINING	3079 ± 874	6.5 ± 2.2	0.9 ± 0.3	1.9 ± 0.2 *
**CON group**	BASELINE	2924 ± 967	5.2 ± 1.9	1.4 ± 0.5	1.4 ± 0.5
*n* = 9	TRAINING	2610 ± 488	5.0 ± 1.3	0.9 ± 0.3 *	1.6 ± 0.4

*: *p* < 0.05 as compared to PRE values.

**Table 2:** Rating perception of effort (RPE) during the 20 km cycling time-trial every 5 km for SL and CON groups in PRE and POST tests.

RPE						
		0	5 km	10 km	15 km	20 km
**SL group**	PRE	9 ± 1.2	14.7 ± 2.3	16.2 ± 1.6	17.3 ± 1.7	19 ± 1.2
	POST	10 ± 2.5	15 ± 2	16 ± 1.6	17.2 ± 1.3	19 ± 1
**CON group**	PRE	10.9 ± 2	14.7 ± 1.7	15.3 ± 2.3	16.2 ± 2	17.7 ± 1.9
	POST	13.1 ± 2.8	14.3 ± 1.7	15 ± 2.4	16 ± 1.7	18 ± 1.7

**Table 3:** Blood analysis sampled before, during (at 1 h) and immediately after (at 2 h) the submaximal test for markers of lipid metabolism (glycerol, non-esterified fatty acid (NEFA)) and catecholamine concentrations.

		**Glycerol (mmol·L^−1^)**			**NEFA (µmol·L^−1^)**		
	**Blood Sampling**	**Before**	**During**	**After**	**Before**	**During**	**After**
**SL group**	PRE	0.02 ± 0.01	0.11 ± 0.06	0.25 ± 0.13	185 ± 115	308 ± 135	610 ± 209
	POST	0.02 ± 0.01	0.07 ± 0.04	0.22 ± 0.1	168 ± 79	229 ± 90	589 ± 213
**CON group**	PRE	0.03 ± 0.01	0.08 ± 0.03	0.21 ± 0.08	153 ± 60	241 ± 148	604 ± 284
	POST	0.03 ± 0.03	0.10 ± 0.05	0.22 ± 0.11	134 ± 59	341 ± 222	699 ± 457
		**Adrenaline (ng·mL^−1^)**			**Noradrenaline (ng·mL^−1^)**		
	**Blood Sampling**	**Before**	**During**	**After**	**Before**	**During**	**After**
**SL group**	PRE	0.10 ± 0.13	0.31 ± 0.25	1.1 ± 0.79	0.93 ± 0.92	4.17 ± 2.1 ^$^	4.6 ± 3.8 ^$^
	POST	0.07 ± 0.10	0.16 ± 0.17	0.73 ± 0.67 *	0.9 ± 0.6	3.8 ± 3.6 *	2.9 ± 2.2 *
**CON group**	PRE	0.18 ± 0.25	0.36 ± 0.1 ^$^	0.27 ± 1.64 ^$^	1.68 ± 1.0	4.13 ± 4.5	7.6 ± 4.4
	POST	0.04 ± 0.04	0.30 ± 0.13	0.48 ± 0.20	5.1 ± 7.2	10.1 ± 7.9	7.3 ± 6.9

^$^ significantly different from PRE before values, *p* < 0.01; * significantly different from POST before values, *p* < 0.05.
